# Intracerebral Haemorrhage in the Top End of the Northern Territory; Risk Factors, Outcomes and the Presence of Cerebral Amyloid Angiopathy in Indigenous Peoples

**DOI:** 10.1111/ajr.70204

**Published:** 2026-07-07

**Authors:** Marianna Diamandopoulos, Alvaro Cervera, Geoffrey A. Donnan

**Affiliations:** ^1^ Flinders University and Royal Darwin Hospital Darwin Northwest Territories Australia; ^2^ Department of Medicine and Neurology Melbourne Brain Centre at Royal Melbourne Hospital Parkville Victoria Australia

**Keywords:** Aboriginal and Torres Strait Islander population, CAA, stroke

## Abstract

**Objective:**

The prevalence of intracranial haemorrhage (ICH) and cerebral amyloid angiopathy (CAA) in the Northern Territory (NT) is unknown. This study aims to improve knowledge of haemorrhagic stroke in the NT, to evaluate the relevance of CAA and examine its association with chronic kidney disease (CKD).

**Methods:**

Retrospective assessment of ICH cases admitted at Royal Darwin Hospital from 2015 to 2019. Epidural, traumatic, or aneurysmal subarachnoid haemorrhages were excluded. We assessed neuroimaging, demographics, kidney function, risk factors, and outcome. We examined differences in the Aboriginal and Torres Strait Islander population, the association of CKD with outcome, and CAA prevalence.

**Results:**

We found 156 cases, mean age 63, 45% women and 34.0% Aboriginal and Torres Strait Islanders. MRI was performed in 39% of patients; 25% had possible/probable CAA. Mortality during admission was 37.2% and 32.7% were dependent. Mortality was independently associated with ICH volume and CKD. Aboriginal and Torres Strait Islander patients were younger, with higher smoking prevalence, diabetes, and CKD, and had lower independence at discharge. However, the prevalence of CAA was not different between groups, and CAA was not associated with CKD.

**Conclusions:**

ICH in the NT has a poor outcome. The main predictors of mortality are CKD and ICH volume. There is no association between CKD and CAA, and CAA is not more prevalent in Aboriginal and Torres Strait Islander patients. CAA may be underestimated due to a lack of MRI, which was rarely performed in patients who died. Therefore, a prospective study is warranted to better characterise CAA in the NT.

## Summary

- What is already known on this subject?
  ○ ICH is a devastating condition associated with poor functional outcome and high mortality.
  ○ CKD and CAA have been identified as risk factors for spontaneous ICH.
  ○ The prevalence of ICH, and particularly CAA‐related ICH, in the Northern Territory's Top End remains undefined.
- What does this study add?
  ○ CKD is associated with a higher mortality after ICH, and it is more common in the Aboriginal and Torres Strait Islander population.
  ○ The main predictors of mortality and dependency after ICH were CKD, ICH volume, and age.
  ○ In our population, CAA was not associated with ethnicity, CKD, or prognosis. However, the percentage of ICH related to CAA is probably underestimated, including its potential relationship to outcome.
  ○ A prospective study to better evaluate CAA in the NT is needed.

## 1 Introduction

Intracranial haemorrhage (ICH) is a devastating condition associated with poor functional outcome and high mortality. It represents 9%–27% of all strokes [1, 2]. Two‐thirds of cases result in death or long‐term disability within 1 year, with survivors having a high risk of recurrent stroke [3]. Mortality remains high, at 36.3% at 3 months and 50.7% during the first year [4].

Major risk factors for spontaneous ICH (sICH) include age, hypertension, and the use of antithrombotic therapy [5, 6, 7, 8]. Other factors include obesity [9], heavy alcohol use [10], tobacco use, stimulant medications, diabetes, and chronic kidney disease (CKD) [11, 12, 13, 14]. Race and ethnicity also appear to be associated with an increased risk of ICH [15].

The signs and symptoms of ICH vary according to the location and size of the haemorrhage, with the location providing insight into the likely cause. The most important diagnostic tool is the CT brain, which can provide further understanding of the aetiology. The most important prognostic factor in intracerebral haemorrhage is the volume of blood on CT at admission [16, 17].

**FIGURE 1:**
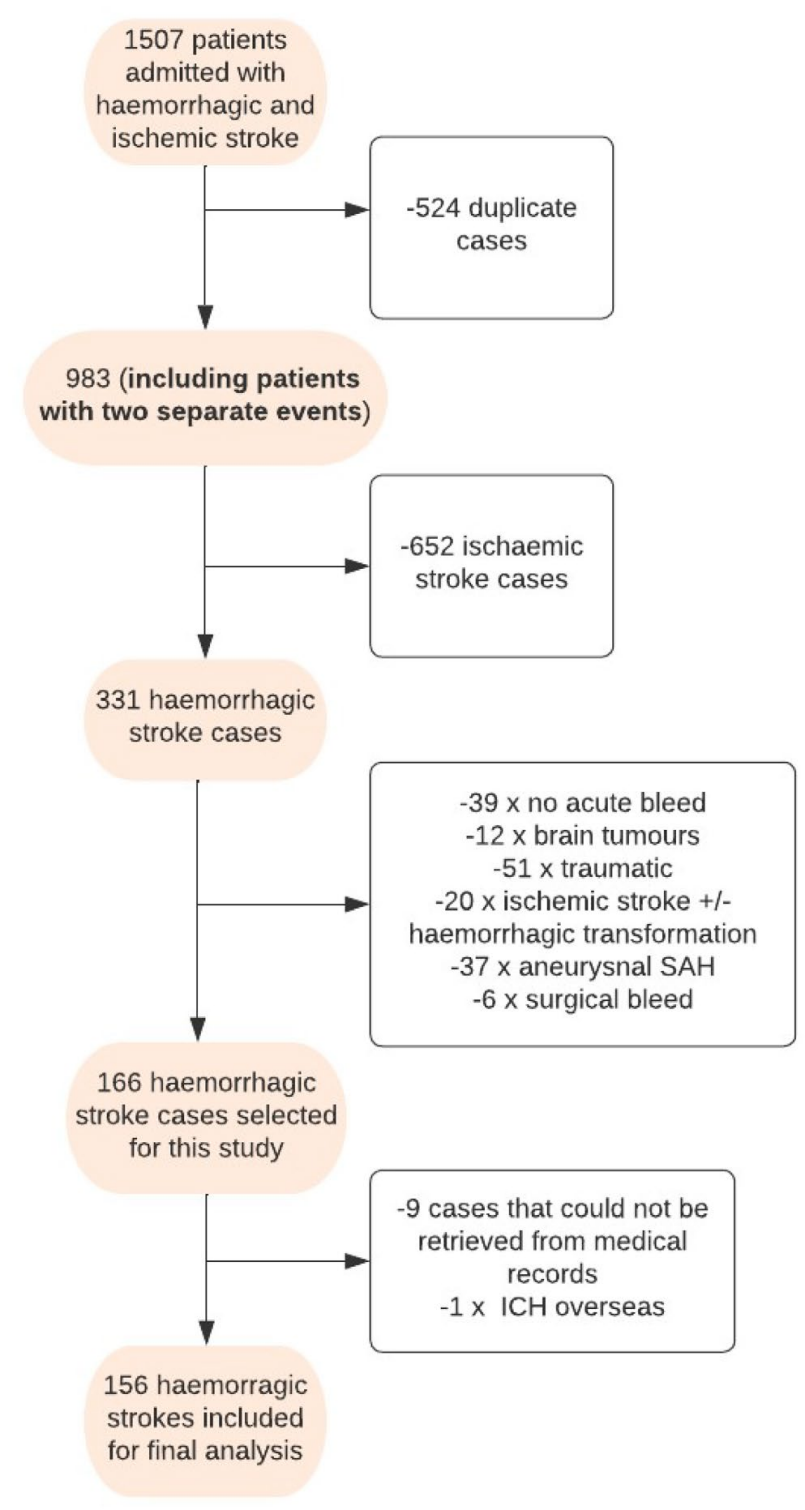
Flowchart of patient inclusion.

Magnetic resonance imaging (MRI) can detect chronic cerebral microbleeds (CMB), which are helpful for the diagnosis of cerebral amyloid angiopathy (CAA) (see Figure 1 in appendix for Boston Criteria) [18, 19]. CAA is an important cause of ICH, mainly in older adults, and has a higher recurrence rate than hypertensive haemorrhage, the most common cause of ICH. CAA is associated with cognitive impairment and may preclude the use of antithrombotic treatment. The pathophysiology of amyloid accumulation is largely unknown; however, it has been proposed that CKD could be a relevant factor in arteriolar amyloid deposition. In addition, CKD is associated with a 43% greater risk of stroke [20] and is related to small vessel disease and CMBs [21, 22], as well as being a strong risk factor for haemorrhagic stroke [22, 23].

Aboriginal and Torres Strait Islander people represent 26.3% of the Northern Territory (NT) population [24]. They are more likely to suffer ICH, and mortality is higher than in non‐Aboriginal patients [25]. The hospital mortality in Darwin was 56% higher in Aboriginal and Torres Strait Islander patients after age adjustment [26]. Furthermore, the prevalence of CKD in our area is the highest in Australia. In the 2014 Australian Bureau of Statistics National Health Survey, the prevalence of CKD was 40% for Indigenous Australians in the NT, compared with 22% for national Indigenous prevalence and 9% for non‐Indigenous Australians [27].

It has been reported that Aboriginal Australians have strokes at a younger age with a greater proportion of ICH [28, 29], increased in‐hospital death rates [30], and they receive a reduced quality of care in hospitals [31].

Despite this, the prevalence of ICH and mainly CAA‐related ICH in the NT's Top End has not been systematically characterised.

This study aims to improve the knowledge of haemorrhagic stroke in the Top End of the NT, as well as the relevance of CAA and its possible association with CKD.

## 2 Methods

### 2.1 Study Design and Participants

This was a hospital‐based retrospective audit analysing all patients admitted to Royal Darwin Hospital (RDH) with ICH from January 2015 to December 2019. The study was approved by the local Human Research Ethics Committee. Given this was a retrospective study, only pre‐existing data were examined and written informed consent was not obtained.

International Classification of Diseases codes (ICD‐10 Version: 2019) were utilised to identify the study cohort. All patients older than 18 years with a diagnosis of ICH, with at least one CT or MRI performed, were identified. Patients who had an epidural bleed, a traumatic bleed (including subarachnoid, contusion and subdural), aneurysmal subarachnoid haemorrhage, or no information regarding risk factors or at least one analysis including kidney function were excluded. Data were extracted from medical records for all cases analysed in this study.

### 2.2 Procedures

Medical records were reviewed to gather information regarding the cause of ICH, demographics, medical history/risk factors, date and time of admission, haemorrhagic stroke onset, initial findings on examination, and kidney function on admission at RDH. The time of onset was set to 00:00 for patients who were unsure about the time of onset of their haemorrhagic stroke, and excessive alcohol consumption was documented as > 12 units/week. Kidney function was assessed using the estimated glomerular filtration rate (eGFR) from admission. CKD was defined as an eGFR of less than 60 mL/min/1.73m^2^.

All radiologic data was reviewed by a neurologist (AC). CT was used to determine the location of the haemorrhage, which was categorised as either subcortical (thalamus, basal ganglia), lobar, brainstem or cerebellum, subarachnoid and subdural. In addition, other findings were documented, such as previous ischaemic stroke, small vessel disease, intraventricular haemorrhage, midline shift and hydrocephalus. CT angiograms were reviewed to exclude aneurysmal ICH or other vascular abnormalities. SWI/T2* MRI sequences, when available, were utilised to identify CMB or focal subarachnoid haemorrhage [32]. With MRI, the diagnosis of small vessel disease was further classified according to Fazekas scale [33]. The diagnosis of CAA was determined using the modified Boston criteria and categorised as no CAA, possible or probable CAA (see Table S3). The intracranial haemorrhage volume in the CT scan was calculated using the ABC/2 formula [34].

### 2.3 Outcomes

The main outcome variables assessed in this study were mortality and dependence at discharge. Dependence was assessed as modified Rankin score (mRS) of 3–5 (see Table S4).

### 2.4 Statistical Analysis

Continuous variables were presented as mean and standard deviation (SD) and categorical variables as absolute numbers and percentages. Comparisons between categorical variables were assessed using Chi‐squared, and for continuous variables, *t*‐test was utilised. Variables associated with mortality and dependency were assessed using logistic regression analysis, including variables with a significant association in the univariate analyses. Statistical analyses were completed using PSPPIRE (Open‐Source Statistical Analysis Package), version 1.4.1. A *p* value of less than 0.05 was considered statistically significant. The Bonferroni correction was used to adjust the significance level when performing multiple tests.

## 3 Results

Of the 1507 patients identified with stroke, we obtained 156 suitable cases with ICH (exclusion process is summarised in Figure 1).

### 3.1 Baseline Characteristics

The baseline characteristics of the 156 patients are shown in Table 1. All included patients had a CT scan on admission. A total of 44.9% of the haemorrhages occurred in the lobar region, 53% occurred in the subcortical region, 18% were subarachnoid, and 15% occurred in the brainstem or cerebellum. The mean intracerebral haemorrhage volume was 36.49 mL (SD 43.52).

**TABLE 1:** Baseline characteristics of the study population.

	% (No.)
	All (*n* = 156)
Female	44.9 (70)
Aboriginal or Torres Strait Islander	34.0 (53)
Current/ex‐smoker	33.9 (53)
Hypertension	64.1 (100)
Diabetes	33.3 (52)
Chronic Kidney Disease	28.2 (44)
Dialysis	10.3 (16)
Excessive alcohol consumption	23.7 (37)
Ischaemic heart disease	30.1 (47)
Previous stroke/TIA	16.0 (25)
Antithrombotic treatment	44.9 (70)
Aspirin	21.2 (33)
Clopidogrel	1.9 (3)
Warfarin	5.8 (9)
Apixaban	1.3 (2)
Rivaroxaban	3.8 (6)
Two antiplatelets	4.5 (7)
Anticoagulant + antiplatelets	3.8 (6)
Other	2.6 (4)
Dementia or cognitive impairment	7.7 (12)
Premorbid mRS	
0	73.7 (115)
1	2.6 (4)
2	13.5 (21)
3	7.1 (11)
4	2.6 (4)
5	0.6 (1)

Abbreviations: TIA, transient ischaemic attack; mRS, modified Rankin score.

### 3.2 Imaging

A brain MRI was completed on 39.1% of patients, and 41% had CMB (mean number 15.31, SD 10.56). According to the modified Boston Criteria, 25% of ICH patients had possible or probable CAA.

### 3.3 Risk Factors and Comparison of Indigenous and Non‐Indigenous Groups

Aboriginal and Torres Strait Islander patients suffered ICH at a younger age (55.44 vs. 68.06 years, *t*‐test *p* < 0.001) (Table 2). In addition, the percentage of smokers, diabetics and CKD was higher in Aboriginal and Torres Strait Islander patients. Hypertensive ICH was the most common aetiology in both Aboriginal and Torres Strait Islander and non‐indigenous patients (47.2% and 36.9%). ICH related to CAA was more frequent in non‐indigenous patients (28.1% vs. 18.8%). However, a brain MRI was available in only 28.3% of Aboriginal and Torres Strait Islander patients compared to 44.7% of non‐indigenous patients (*p* = 0.047) (Table 2). Further differences between Aboriginal and Torres Strait Islander patients and non‐indigenous patients for other diagnoses attributed to ICH are listed in Table 2.

**TABLE 2:** Comparing variables of the study population according to ethnicity.

	Aboriginal and Torres Strait Islander	Non‐indigenous	*p* (2‐tailed)
	% (no.) or mean (standard deviation)	% (no.) or mean (standard deviation)	
Risk factors			
Female	47.2 (25)	43.7 (45)	0.679
Age	55.44 (11.92)	68.06 (14.19)	0.000*
Weight	68.75 (16.35)	78.35 (19.59)	0.005
Current/ex‐smoker	38.4 (20)	32.1 (33)	0.021
Hypertension	67.9 (36)	62.1 (64)	0.475
Diabetes	54.7 (29)	22.5 (23)	0.000*
CKD	58.5 (31)	12.7 (13)	0.000*
Dialysis	26.4 (14)	2.0 (2)	0.000*
Excessive alcohol consumption	37.7 (20)	16.5 (17)	0.003
IHD	30.2 (16)	30.4 (31)	0.979
Previous stroke or TIA	13.2 (7)	17.6 (18)	0.476
Antithrombotic treatment	47.2 (25)	43.7 (45)	0.679
Dementia or cognitive impairment	3.8 (2)	9.7 (10)	0.188
Findings on examination during admission			
Haemoglobin (g/L)	115.98 (25.01)	133.58 (22.17)	0.000*
Capillary blood glucose (mmol/L)	9.19 (3.46)	8.01 (3.15)	0.042
Creatinine	235.92 (237.87)	97.30 (88.82)	0.000*
eGFR	54.02 (35.97)	71.93 (22.75)	0.000*
SBP	173.29 (40.52)	162.87 (35.14)	0.109
DBP	89.77 (20.93)	87.22 (21.49)	0.499
Radiology characteristics			
Time CT	239.94 (447.22)	142.05 (191.90)	0.058
Onset CT	1107.70 (1284.88)	1321.58 (2201.64)	0.515
Location of haemorrhage			
Subcortical brain (thalamus, basal ganglia)	39.6 (21)	31.1 (32)	0.591
Lobar	37.7 (20)	48.5 (50)	
Brainstem or cerebellum	11.3 (6)	8.7 (9)	
Subarachnoid	11.3 (6)	11.7 (12)	
Previous ischemic stroke on CT	11.3 (6)	12.6 (13)	0.814
Small vessel disease on CT	18.9 (10)	35.9 (37)	0.028
Intraventricular haemorrhage	45.3 (24)	37.9 (39)	0.371
Midline shift	58.5 (31)	47.6 (49)	0.196
Hydrocephalus	45.3 (24)	35.0 (36)	0.209
ICH volume	37.72 (35.93)	35.86 (47.14)	0.812
MRI done	28.3 (15)	44.7 (46)	0.047
Presence of small vessel disease on MRI	73.3 (11)	63.0 (29)	0.466
Enlarged perivascular spaces on MRI	53.3 (8)	50.0 (23)	0.823
Focal SAH on MRI	20.0 (3)	34.8 (16)	0.283
Microbleeds on MRI Number	53.3 (8)	37.0 (17)	0.263
Diagnosis according to Boston Criteria	17.13 (9.93)	14.50 (11.01)	0.569
Possible or probable CAA	18.8 (10)	28.1 (29)	0.432
Outcomes			
mRS discharge			
0–2	20.8 (11)	35 (36)	0.109
6	41.5 (22)	35.0 (36)	
Etiologic diagnosis			
Hypertensive ICH	47.2 (25)	36.9 (38)	0.016
CAA related ICH	17.0 (9)	23.3 (24)	
Anticoagulant associated ICH	11.3 (6)	14.6 (15)	
Spontaneous SDH or SAH	7.5 (4)	9.7 (10)	
ICH associated to underlying lesion	1.9 (1)	12.6 (13)	
Other	15.1 (8)	2.9 (3)	

*Note:* A Bonferroni correction was applied for 37 comparisons with a corrected significance level of *p* < 0.00135.

Abbreviations: CAA, cerebral amyloid angiopathy; CKD, chronic kidney disease; DBP, diastolic blood pressure; eGFR, estimated glomerular filtration rate; ICH, intracerebral haemorrhage; IHD, ischaemic heart disease; MRI, magnetic resonance imaging; mRS, modified Rankin score; SAH, subarachnoid haemorrhage; SBP, systolic blood pressure; TIA, transient ischaemic attack.

* Significant after Bonferroni correction.

### 3.4 Outcome

Mortality at discharge was 37.2%, and only 30.1% of patients were independent at discharge. Only 3.4% of patients who died during admission underwent a brain MRI, compared to 60.2% of survivors. Independence at discharge was non‐significantly more frequent in non‐indigenous patients (35.0% vs. 20.8%, *p* = 0.067) compared to Aboriginal and Torres Strait Islander patients. Patients who died after an ICH were older (66.41 vs. 62.21, *t*‐test 0.084) and had a higher ICH volume (67.43 mL vs. 15.37 mL, *p* < 0.01) (Table S2). The location and etiologic diagnosis of the haemorrhage were also statistically associated with mortality and independence (data not shown). Mortality was very high in patients who suffered a haemorrhage in the brainstem or cerebellum (73.3%), and in patients with ICH associated with anticoagulation (81.0%). Conversely, there was a higher percentage of patients who achieved independence after a non‐aneurysmal subarachnoid haemorrhage (77.8%). Mortality was also associated with a higher systolic blood pressure on admission (175.38 mmHg vs. 160.74 mmHg, *t*‐test *p* = 0.019) and a higher capillary blood glucose (9.39 vs. 7.75, *p* = 0.003) (Table S2). Mortality among patients with CKD was 59.1%, and only 11.4% were independent at discharge (Table 3). Patients who died had a lower eGFR at admission (57.30 vs. 73.81, *t*‐test *p* = 0.006) (Table S2).

**TABLE 3:** Risk factors and prognostic variables according to the presence of chronic kidney disease.

	Patients with CKD mean (standard deviation) or %(no.)	Patients without CKD mean (standard deviation) or %(no.)	*p* (2 tailed)
Age	63.34 (14.17)	63.88 (15.02)	0.836
Aboriginal or Torres Strait Islander	70.5 (31)	19.9 (22)	0.000*
Diabetes	65.9 (29)	20.7 (23)	0.000*
Creatinine	304.05 (248.29)	81.47 (41.18)	0.000*
SBP	185.36 (38.36)	158.82 (34.15)	0.000*
DBP	92.93 (21.16)	86.19 (21.21)	0.086
ICH volume	53.49 (48.10)	28.73 (39.33)	0.002*
Number of microbleeds	21.00 (9.38)	13.95 (10.58)	0.185
Diagnosis of possible and probable CAA	29.6 (13)	23.4 (26)	0.265
Mortality at discharge	59.1 (26)	27.9 (31)	0.000*
mRS 0 to 2	11.4 (5)	37.8 (42)	0.001*

*Note:* A Bonferroni correction was applied for 11 comparisons with a corrected significance level of *p* < 0.00454.

Abbreviations: CAA, cerebral amyloid angiopathy; DBP, diastolic blood pressure; ICH, intracerebral haemorrhage; mRS, modified Rankin score; SBP, systolic blood pressure.

* Significant after Bonferroni correction.

In a logistic regression analysis, CKD (OR = 5.47, *p* = 0.011), ICH volume (OR = 1.05, *p* = 0.000) and a diagnosis of possible or probable CAA (OR = 0.15, *p* = 0.006) were found to be the main independent predictors of mortality (Table 4). Similarly, the main predictors of functional independence were ICH volume (OR = 0.85, *p* = 0.000) and age (OR = 0.94, *p* = 0.013) (Table 5).

**TABLE 4:** Logistic regression assessing variables associated with mortality.

	OR	95% CI	*p*
CKD	5.47	1.47–20.38	0.011*
ICH volume	1.05	1.03–1.07	0.000***
CAA possible and probable	0.15	0.04–0.58	0.006**
Aboriginal and Torres Strait Islander	0.32	0.09–1.11	0.072

Abbreviations: CAA, cerebral amyloid angiopathy; CKD, chronic kidney disease; ICH, intracerebral haemorrhage.

* *p* ≤ 0.05.

** *p* ≤ 0.01.

*** *p* ≤ 0.001.

**TABLE 5:** Logistic regression assessing variables associated with independence (mRS 0–2).

	OR	95% CI		*p*
CKD	0.14	0.02–0.89		0.038*
ICH volume	0.85	0.79–0.93		0.000***
CAA possible and probable	2.32	0.67–8.04		0.183
Aboriginal and Torres Strait Islander	0.89	0.18–4.35		0.889
Age	0.94	0.89–0.99		0.013*
IHD	3.15	0.94–10.57		0.063

Abbreviations: CAA, cerebral amyloid angiopathy; CKD, chronic kidney disease; ICH, intracerebral haemorrhage; IHD, ischaemic heart disease.

* *p* ≤ 0.05.

** *p* ≤ 0.01.

*** *p* ≤ 0.001.

## 4 Discussion

Our study found that ICH in the NT is associated with poor outcomes, which appear to be worse in Aboriginal and Torres Strait Islander patients. One of the main risk factors for a worse outcome is CKD, which was more prevalent in this group. However, we could not find an association between CAA and CKD or a higher prevalence of CAA in Indigenous patients.

ICH was slightly more frequent than expected in the Aboriginal and Torres Strait Islander population, as they represented 34% of our cohort (Table 1), compared to 26% of the population in the NT. In addition, Aboriginal and Torres Strait Islanders suffered an ICH at a younger age and were more frequently smokers, diabetics, had CKD, or excessively high alcohol consumption. Regarding kidney function, Aboriginal and Torres Strait Islander patients had a higher mean creatinine at admission and a lower eGFR. The etiological diagnosis was also statistically different between ethnicities. Aboriginal and Torres Strait Islander patients were more likely to suffer hypertensive ICH compared to non‐indigenous patients, possibly attributable to a higher burden of CKD. Despite these differences in comorbidities, mortality at discharge was not different between Indigenous and non‐Indigenous patients. However, there was a trend toward worse outcomes, with more patients dependent for daily activities at discharge in the Aboriginal and Torres Strait Islander group.

It was interesting to note that fewer Aboriginal and Torres Strait Islander patients had a brain MRI during admission, although the cause of this difference is not known. A brain MRI was rarely performed in patients who died during admission, and Aboriginal and Torres Strait islander patients had a higher percentage of brainstem or cerebellum bleeds as well as lobar bleeds, compared to non‐indigenous patients. Therefore, patients may have died before receiving an MRI, as both types of haemorrhage were associated with high mortality. As MRI is an important tool to diagnose the aetiology of ICH, mainly CAA, this could represent a significant bias, potentially underestimating the incidence of CAA and its relation to outcome.

The majority of intracerebral haemorrhages occurred in the lobar region, followed by the subcortical region. Hypertension was the most common risk factor. In a meta‐analysis of 28 studies, it was revealed that hypertension was twice as common in patients with deep intracerebral haemorrhage as in patients with lobar intracerebral haemorrhage [35]. Since CAA is one important cause of lobar bleeds, this finding suggests that our population may have a higher number of CAA‐related ICH. Lobar ICH is a devastating disease with a high risk of recurrence and mortality [5, 36]. This is consistent with the data obtained from our study, as lobar haemorrhage was associated with a high mortality. Despite this, we could not find an association between CAA and outcome. However, a brain MRI is needed to diagnose CAA, and this was not available in the majority of patients in our cohort with poor outcome.

Independence was more frequent in younger patients who suffered an ICH (Table S1). Age was not significantly associated with mortality in our study. A multivariate regression analysis identified ICH volume as a risk factor for mortality and dependence [16], which is consistent with our results. In addition, we found that ICH related to anticoagulation was associated with a very high risk of death. A case–control study of patients in Denmark showed that anticoagulant therapy, particularly warfarin, is associated with an increased risk of ICH, whereas the risk with antiplatelet monotherapy appears to be minimal [37]. Mortality was also associated with a higher systolic and diastolic blood pressure on admission and a higher capillary blood glucose. Stress hyperglycaemia mediated by cortisol and norepinephrine release is more relevant than the effect of diabetes [38].

Our study confirmed that there is a high burden of kidney disease within our population, particularly in Aboriginal and Torres Strait Islander patients. In addition, mortality was very high, and only a small percentage was independent on follow‐up. One explanation is a higher ICH volume in CKD patients, which has been shown to have a worse prognosis after ICH [16]. A significant proportion of CKD patients also suffered from diabetes. In the NT, the main risk factor for CKD in Aboriginal and Torres Strait Islanders is diabetes [39]. Furthermore, CKD was associated with higher SBP at admission.

This was supported in a logistic regression analysis showing that CKD was an independent risk factor for mortality (OR = 5.47, 95% CI = 1.47–20.38, *p* value = 0.011) and was associated with a lower rate of independence (OR = 0.14, 95% CI = 0.02–0.89, *p* value = 0.038) (Table 4 and Table 5). CKD is an emerging independent risk factor not only for ischaemic and haemorrhagic stroke, but also for cerebral small vessel disease [40]. There are several possible explanations for why CKD acts as a significant risk factor for ICH and a marker of mortality. One explanation is the association of CKD with small vessel microangiopathy, as the induction of endothelial permeability due to high volume blood flow to these low‐resistance‐end arterial organs (brain and kidney) is the primary underlying pathology for both diseases [41]. Despite this pathophysiology hypothesis of the association between CKD and CAA, our study showed that CAA was not more prevalent in CKD. CKD also causes chronic inflammation, oxidative stress, elevated levels of asymmetric dimethylarginine, thrombogenic factors and uremic toxins, which lead to cerebrovascular injury and endothelial dysfunction [40].

It was expected that CAA would be more prevalent in Aboriginal and Torres Strait Islanders due to the high disease burden of CKD, which was not the case in our study. Although this concept is supported by literature, there were a number of limitations within this study that may explain this. First, our sample size was small, with only 21 patients diagnosed as having probable CAA and 18 patients diagnosed as having possible CAA out of 156 patients presenting to RDH with ICH. In addition, a significant percentage of patients did not have a brain MRI, especially those who died. Consequently, the number of CAA was likely underestimated. Another limitation was the frequent incomplete information when reviewing medical records due to the retrospective nature of this study. However, this is the larger study assessing ICH in our environment and offers insight into risk factors and outcomes that can be useful for future studies. One potential controversy is the worse outcome in Aboriginal and Torres Strait Islanders, and why these patients had less frequent access to a brain MRI, which is an important diagnostic tool in this condition.

## 5 Conclusion

In conclusion, spontaneous ICH is a devastating condition in the NT with high mortality and dependency. The main predictors of mortality and dependency were CKD, ICH volume and age. CKD is also much more common in the Aboriginal and Torres Strait Islander population. CAA is a common cause of ICH in the Top End. However, our study did not find an association with ethnicity or CKD, and it was not associated with prognosis. Nevertheless, mortality was high, and these cases rarely had an MRI to guide the aetiologic diagnosis. Consequently, it is likely that the prevalence of CAA is underestimated, as well as its relationship to outcome, highlighting the need for a prospective study to better characterise CAA in the NT.

## Author Contributions

**Marianna Diamandopoulos:** conceptualization, investigation, writing – original draft, methodology, validation, software, formal analysis, resources, writing – review and editing, project administration, supervision, data curation. **Alvaro Cervera:** conceptualization, investigation, writing – original draft, methodology, validation, writing – review and editing, software, formal analysis, project administration, data curation, supervision, resources. **Geoffrey A. Donnan:** writing – review and editing, supervision.

## Funding

The authors have nothing to report.

## Ethics Statement

The study was approved by the Human Research Ethics Committee of the Northern Territory Department of Health and Menzies School of Health Research (HREC 2020‐3645).

## Supporting information


**Table S1:** Prognostic factors for independence (mRS 0 to 2) at discharge of patients with sICH.
**Table S2:** Prognostic factors for mortality (mRS 6) at discharge of patients with sICH.
**Table S3:** Modified Boston Criteria for diagnosis of CAA [35].
**Table S4:** Modified Rankin Score.

## Data Availability

The data that support the findings of this study are available on request from the corresponding author. The data are not publicly available due to privacy or ethical restrictions.
