# Supplementary material for: Intracerebral Haemorrhage in the Top End of the Northern Territory; Risk Factors, Outcomes and the Presence of Cerebral Amyloid Angiopathy in Indigenous Peoples
Source: Aust J Rural Health. 2026 Jul 7;34(4):e70204. doi: 10.1111/ajr.70204 (PMC13342770; doi:10.1111/ajr.70204)
Supplement: Supplementary file 1 — Table S1: Prognostic factors for independence (mRS 0 to 2) at discharge of patients with sICH. Table S2: Prognostic factors for mortality (mRS 6) at discharge of patients with sICH. Table S3: Modified Boston Criteria for diagnosis of CAA [35]. Table S4: Modified Rankin Score. [file AJR-34-0-s001.docx]

Supplementary Information

**Supplementary Table 1**: *Prognostic factors for independence (mRS 0 to 2) at discharge of patients with sICH*

|  | **mRS of 0-2**  mean (standard deviation) | **mRS of 3-6**  mean (standard deviation) | **P-value (2-tailed)** |
| --- | --- | --- | --- |
| Age  Capillary blood glucose (mmol/L)  Creatinine  eGFR  SBP  DBP  ICH volume | 59.81 (14.69)  7.18 (3.00)  104.53 (93.45)  73.81 (24.15)  148.54 (27.13)  82.27 (13.83)  5.41 (4.92) | 65.48 (14.44)  8.90 (3.28)  163.41 (192.09)  62.14 (30.60)  174.02 (38.36)  90.54 (23.43)  46.65 (45.70) | 0.027*  0.003**  0.048*  0.022*  0.000***  0.029*  0.000*** |

Abbreviations: eGFR, estimated glomerular filtration rate; SBP, systolic blood pressure; DBP, diastolic blood pressure; ICH, intracerebral haemorrhage.

Note: *p$\leq0.05$, **p$\leq0.01$, ***p$\leq0.001$

**Supplementary Table 2:**. *Prognostic factors for mortality (mRS 6) at discharge of patients with sICH*

|  | **mRS of 6 at discharge**  mean (standard deviation) | **mRS of 0-5 at discharge**  mean (standard deviation) | **P-value (2-tailed)** |
| --- | --- | --- | --- |
| Age  Capillary blood glucose (mmol/L)  Creatinine  eGFR  SBP  DBP  ICH volume | 66.41 (16.12)  9.39 (3.41)  178.43 (195.73)  57.30 (32.31)  175.38 (45.15)  92.43 (26.76)  67.43 (50.18) | 62.21 (13.64)  7.75 (3.05)  126.214 (150.87)  70.59 (26.21)  160.74 (30.37)  85.35 (16.71)  15.37 (19.48) | 0.084  0.003**  0.088  0.010**  0.034*  0.079  0.000*** |

Abbreviations: eGFR, estimated glomerular filtration rate; SBP, systolic blood pressure; DBP, diastolic blood pressure; ICH, intracerebral haemorrhage.

Note: *p$\leq0.05$, **p$\leq0.01$, ***p$\leq0.001$

**Supplementary Table 3**: *Modified Boston Criteria for diagnosis of CAA.* [35]

1. Definite CAA

Full post-mortem examination demonstrating:

Lobar, cortical, or cortical-subcortical hemorrhage

Severe CAA with vasculopathy

Absence of other diagnostic lesion

2. Probable CAA with supporting pathology

Clinical data and pathologic tissue (evacuated hematoma or cortical biopsy) demonstrating:

Lobar, cortical, or cortical-subcortical hemorrhage (including ICH, CMB, or cSS)

Some degree of CAA in specimen

Absence of other diagnostic lesion

3. Probable CAA

Clinical data and MRI or CT demonstrating:

Multiple hemorrhages (ICH, CMB) restricted to lobar, cortical, or cortical-subcortical regions (cerebellar hemorrhage allowed), OR single lobar, cortical, or cortical-subcortical hemorrhage and cSS (focal or disseminated)

Age≥55 years

Absence of other cause of hemorrhage*

4. Possible CAA

Clinical data and MRI or CT demonstrating:

Single lobar, cortical, or cortical-subcortical ICH, CMB, or cSS (focal or disseminated)

Age ≥55 years

Absence of other cause of hemorrhage*

Greenberg, S.M. and A. Charidimou, Diagnosis of Cerebral Amyloid Angiopathy: Evolution of the Boston Criteria. Stroke (1970), 2018. 49(2): p. 491-497.

**Supplementary Table 4:** *Modified Rankin Score*.


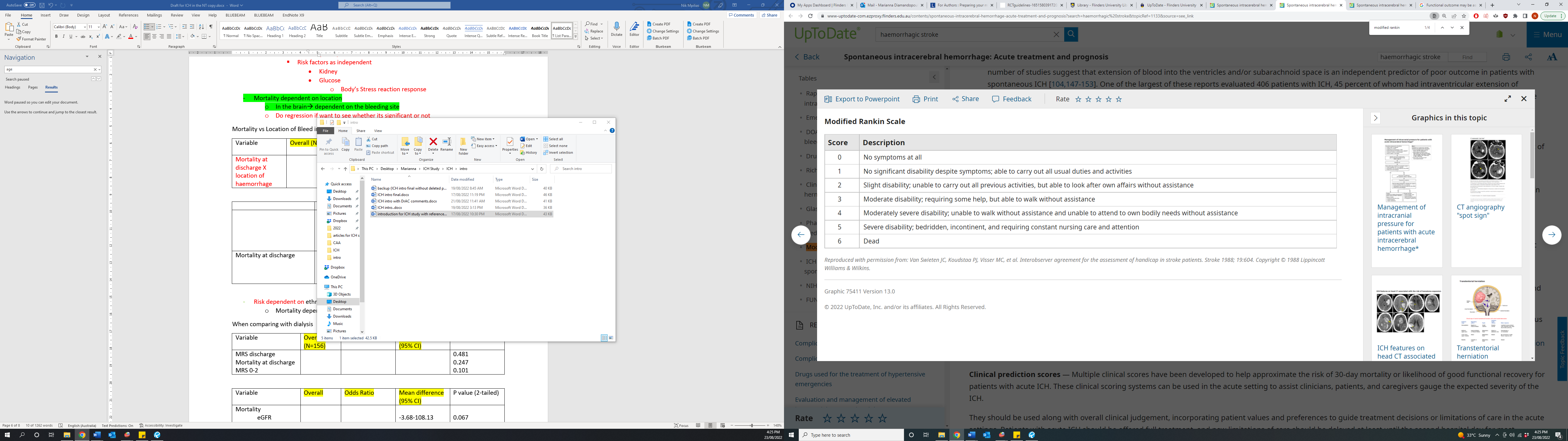


Rankin, J., Cerebral Vascular Accidents in Patients over the Age of 60: II. Prognosis. Scottish Medical Journal, 1957. 2(5): p. 200-215.
